# NADH Oxidase Functions as an Adhesin in *Streptococcus pneumoniae* and Elicits a Protective Immune Response in Mice

**DOI:** 10.1371/journal.pone.0061128

**Published:** 2013-04-08

**Authors:** Lena Muchnik, Asad Adawi, Ariel Ohayon, Shahar Dotan, Itai Malka, Shalhevet Azriel, Marilou Shagan, Maxim Portnoi, Daniel Kafka, Hannie Nahmani, Angel Porgador, Johnatan M. Gershoni, Donald A. Morrison, Andrea Mitchell, Michael Tal, Ronald Ellis, Ron Dagan, Yaffa Mizrachi Nebenzahl

**Affiliations:** 1 Pediatric Infectious Disease Unit, Soroka University Medical Center, Beer Sheva, Israel; 2 The Shraga Segal Department of Microbiology and Immunology, Faculty of Health Sciences, Ben Gurion University of the Negev, Beer Sheva, Israel; 3 Protea Vaccine Technologies and NasVax Ltd., Ness Ziona, Israel; 4 Department of Cell Research and Immunology, Tel Aviv University, Tel Aviv, Israel; 5 Department of Biological Sciences, University of Illinois at Chicago, Chicago, Illinois, United States of America; 6 School of Immunity and Infection, College of Medical and Dental Sciences, University of Birmingham, Edgbaston, United Kingdom; Instituto Butantan, Brazil

## Abstract

The initial event in disease caused by *S. pneumoniae* is adhesion of the bacterium to respiratory epithelial cells, mediated by surface expressed molecules including cell-wall proteins. NADH oxidase (NOX), which reduces free oxygen to water in the cytoplasm, was identified in a non-lectin enriched pneumococcal cell-wall fraction. Recombinant NOX (rNOX) was screened with sera obtained longitudinally from children and demonstrated age-dependent immunogenicity. NOX ablation in *S. pneumoniae* significantly reduced bacterial adhesion to A549 epithelial cells *in vitro* and their virulence in the intranasal or intraperitoneal challenge models in mice, compared to the parental strain. Supplementation of Δ*nox* WU2 with the *nox* gene restored its virulence. Saturation of A549 target cells with rNOX or neutralization of cell-wall residing NOX using anti-rNOX antiserum decreased adhesion to A549 cells. rNOX-binding phages inhibited bacterial adhesion. Moreover, peptides derived from the human proteins contactin 4, chondroitin 4 sulfotraferase and laminin5, homologous to the insert peptides in the neutralizing phages, inhibited bacterial adhesion to the A549 cells. Furthermore, rNOX immunization of mice elicited a protective immune response to intranasal or intraperitoneal *S. pneumoniae* challenge, whereas pneumococcal virulence was neutralized by anti-rNOX antiserum prior to intraperitoneal challenge. Our results suggest that in addition to its enzymatic activity, NOX contributes to *S. pneumoniae* virulence as a putative adhesin and thus peptides derived from its target molecules may be considered for the treatment of pneumococcal infections. Finally, rNOX elicited a protective immune response in both aerobic and anaerobic environments, which renders NOX a candidate for future pneumococcal vaccine.

## Introduction

*Streptococcus pneumoniae* is a commensal pathogen that can cause clinical diseases including otitis media, pneumonia and life-threatening invasive diseases such as bacteremia and meningitis [1]. *S. pneumoniae* is responsible for the annual mortality of over 1.5 million infants worldwide [2]–[4]. This high mortality rate and the substantial increase in *S. pneumoniae* antibiotic resistance have encouraged the search for novel preventive and therapeutic measures [1].

Capsular polysaccharide-based vaccines, which elicit serotype-specific immune responses, were found to be ineffective in young children, who bear the heaviest burden of *S. pneumoniae* infections. Pneumococcal conjugate vaccines (PCV) were found to be effective in young children and led to a substantial decrease in carriage and disease caused by capsular strains covered by the vaccine. However, vaccination with 7-valent PCV led to an increase of carriage and disease caused by capsular strains not covered by the vaccine [4]. Moreover, nasopharyngeal pneumococcal carriage shortly before PCV administration causes serotype hyposensitivity in early infancy [5].

Understanding the sequential molecular interactions of *S. pneumoniae* with its human host may lead to the identification of molecules crucial for disease development [6]. Recently, two types of pili were identified in *S. pneumoniae*. The first is an oligomeric appendage coded by the *rlrA* operon [7] and the second is encoded by the pilus islet named PI-2 [8]. Additional *S. pneumoniae* cell-wall and membrane-residing adhesins have also been identified. Among these are phosphorylcholine, which binds to the platelet activating factor receptor (PAF-R) [9], the lipoprotein PsaA [10], which binds to the E-Cadherin receptor [11] and Pav-A protein which binds to the extracellular matrix protein fibronectin, which in turn can bind an integrin receptor [12]. CbpA (also known as SpsA or PspC) [13] is considered an invasin, and upon binding to either the polymeric immunoglobulin receptor or to secretory IgA facilitates the translocation of *S. pneumoniae* through the mucosal cell layer [14]–[16].

NADH oxidase (NOX) facilitates the reduction of molecular oxygen into water and thus is most important to the anaerobic *S. pneumoniae*, which lacks catalase [17]. Deletion or mutation of the *nox* gene in *S. agalactiae* and in *S. pneumoniae* was accompanied by growth arrest under aerobic but not anaerobic conditions *in vitro* [18], [19].

We found that NOX localizes to *S. pneumoniae* cell-wall [20], where it may be associated with functions other than those attributed to its oxygen reducing activity. In the present study, we observed that NOX functions as an adhesin and identified putative host target molecules. We then further evaluated its vaccine potential and found that recombinant NOX (rNOX) elicits protective immune response in both aerobic and anaerobic environments.

## Materials and Methods

### Ethics Statement

All human studies and protocol revisions were approved by the Helsinki Ethics Committee of the Soroka University Medical Center, Beer Sheva, Israel (Permit number: 10391). We obtained written informed consent from all participants. We obtained a written informed consent from the next of kin, carers or guardians on behalf of the minors/children participants involved in this study. The Helsinki Ethics Committee approved the consent procedure. This study was carried out in strict accordance with the recommendations in the Guide for the Care and Use of Laboratory Animals of the National Institutes of Health. The protocol was approved by the Institutional Animal Care and Use Committee of the Ben-Gurion University of the Negev, Beer Sheva, Israel (Permit number: 53.08.08).

### Reagents

Unless otherwise stated, all chemicals and biochemicals of highest purity available were purchased from Sigma-Aldrich (St. Louis, MO).

### Bacterial strains and growth conditions

Two genetically unrelated encapsulated *S. pneumoniae* strains were used, serotype 2 strain D39 [21] and serotype 3 strain WU2 [22] together with their unencapsulated derivatives, strain R6 (ATCC, Rockville MD) and strain 3.8DW [23], [24], respectively. Pneumococci were grown in THY or on blood agar plates as previously described [25]. Two *Escherichia coli* strains were used, *E. coli* DH5α UltraMAX (DH5α; Invitrogen Corp, Carlsbad, CA) and *E. coli* BL21(DE3)pLysS (BL21; Promega Corp, Madison, WI) and were grown in lysogeny broth (LB).

### Mice

Seven-week-old BALB/cOlaHsd (BALB/c) female mice (Harlan Laboratories, Jerusalem Israel) or seven-week-old CBA/CaHN-*Btk^xid^*/J (CBA/N) mice (Jackson Laboratories, Bar Harbor, ME) were used in this study.

### Cloning, expression and purification of recombinant proteins

The *nox* gene (R6 strain locus AAL00127; protein accession number NP_358916.1) was amplified from *S. pneumoniae* strain R6 genomic DNA by polymerase chain reaction (PCR) with the following primers: **Forward**: ′5 –TGG ATC CAT GAG TAA AAT CGT TGT AGTC –3′, **Reverse:** 5′- TGA GCT CTT ATT TTT CAG CCG TAA GGG-3′, supplemented with restriction enzyme sequences of *Bam*H1 on the 3′ end and *Sac*1 on the 5′ end. The amplified product was cloned into the pHAT expression vector (BD Biosciences Clontech, Palo Alto, CA), and transformed into DH5α cells. The vector was purified and transformed into BL21cells. The expression of the protein and its purification were performed as previously described [25]. The tagged-purified protein was resolved by SDS-PAGE demonstrating the predicted size of NOX fused to the HAT tag (∼53 kDa) with 95% purity.

To verify that the function in adhesion and the immunogenicity observed for the tagged protein are not affected by the HAT-tag, an untagged protein was cloned and purified. Although the published sequences of NOX in the R6 and the TIGR4 strains are highly homologous, the gene for the preparation of the untagged rNOX protein was amplified from the *S. pneumoniae* TIGR 4 strain (locus AAK75563; protein NP_345923; ATCC, Rockville, MD) using the same primers as those used to amplify the gene from R6, except that the *Nde*I and *Bpu1102*I restriction enzyme sites were used instead of *Bam*H1 and *Sac*1, and cloned into pET30a(+). Digestion of the vector with NdeI and Bpu1102I enzymes prior to ligation will remove all tags from the vector. The protein expression was induced with IPTG, and the protein residing in inclusion bodies was solubilized with Tris buffer containing 4.5 M urea and 0.1 mM cysteine and brought to pH 11.3. The protein was refolded by dialysis against 10 mM Tris buffer at pH 8.0. The protein was purified using Q-Sepharose column. The purified protein was dialyzed against NaHCO_3_ and lyophilized.

The rNOX protein sequence was verified by MALDI-TOF analysis as previously described using the Bruker Reflex-IV mass spectrometer (Bruker-Daltonik, Bremen, Germany) [20]. Alignment was performed using both the Mascot software package (Matrix Science Ltd., UK, http://www.matrixscience.com) and Profound program provided by the Rockefeller University.

### Preparation of rabbit anti-rNOX antiserum

Three-month-old white albino rabbits (Harlan Laboratories, Israel) were immunized intramuscularly with 200 µg HAT-rNOX emulsified with CFA (ratio 1∶1) for the first immunization or IFA for booster immunizations. Two weeks after the last immunization, rabbits were exsanguinated and sera were prepared.

### Age-dependent antigenicity

In an attempt to analyze the antibody levels against rNOX in children at different ages, rNOX (10 ng) was separated by SDS-polyacrylamide-gel electrophoresis (SDS-PAGE) and transferred to nitrocellulose membranes. The lanes on the membranes were cut and probed with human sera. Sera were collected longitudinally at 18, 30 and 42 months of age from healthy children attending day care centers. In addition, sera obtained from different children at 7, 12, 24 and 38 months of age and sera obtained from random mothers were also analyzed. Following blocking (skim milk 5%), the strips were incubated with the human sera (at 1∶20 dilution) overnight. Except for the origin of the sera in each of these two experiments (which were repeated twice each), all other parameters were kept the same. The human sera were detected with goat anti-human IgG-HRP antibody (Santa Cruz Biotechnologies Inc., Santa Cruz, CA). All the strips from each experiment were developed together for 20 seconds (Microchemi 4.2; DNR, Jerusalem, Israel). Nasopharyngeal (NP) swabs were taken from the children bimonthly, and the episodes of carriage of different *S. pneumoniae* serotypes were documented as previously described [26].

### Immunoblot analysis of *S. pneumoniae* cell-wall proteins

*S. pneumoniae* cell-wall fraction proteins were separated by SDS-PAGE under reduced conditions and transferred to nitrocellulose membranes (Bio-Rad Laboratories Inc, Carlsbad, CA). The identity of NOX was confirmed by immunoblot analysis using rabbit anti-HAT-NOX antiserum that was detected with peroxidase-conjugated AffiniPure F(ab′)_2_ fragment goat anti-rabbit IgG (H+L; Jackson Laboratories, Bar Harbor, ME).

### Creation of nox null mutant bacteria

To create bacteria with a null mutation at the *nox* gene, the gene was replaced with an erythromycin (Em^r^) resistance gene using homologous recombination as previously described [27]. The downstream (468 bp) and the upstream (664 bp) fragments of *nox* were amplified from genomic DNA of *S. pneumoniae* strain R6. The downwing was amplified using forward primer IM1: CTTCAACAAAATGCCCGATT and reverse primer IM2: gctgggcCCATACAACTACATCACAATGGCT which contains the *Apa*I terminus. The upwing was amplified with the forward primer IM3: ccgcggatccTTCCCCATTTATCCTGTTTTCAAGCC and reverse primer IM4: ATCCGCACTGTATCCTTTGG containing the *Bam*HI terminus. An 870 bp fragment containing an *ermAM* gene encoding resistance to erythromycin was amplified from DAM amplicon using forward primers IM5: CCGCGGATCCAGTCGGCAGCGACTCATAGAAT and reverse primer DAM212: CCGGGCCCAAAATTTGTTTGAT containing *Apa*I and *Bam*HI termini, respectively. The PCR products were digested with the corresponding restriction nucleases, purified and ligated to produce a 2 kbp cassette. The cassette was transformed into *S. pneumoniae* strain R6 and serotype 3 strain WU2 bacteria following treatment with competence stimulating factor 1 (CSP1) [27]. Transformants were selected for Em^r^. NOX was deleted in the unencapsulated serotype 2 derived R6 strain and in serotype 3 strain WU2 and the deletion was verified by PCR and immunoblotting. (Figure S1 in File S1).

### Complementation of Δnox WU2 with the native nox gene

The *nox* gene was amplified using the following primers: Forward: ′5 –GGT GGT ACT AGT ATG AGT AA A ATC GTT GTA GTC –3′, supplemented with the *Spe1* restriction site; Reverse: 5′- GGT GGT CTG CAG TTA TTT TTC AGC CG T AAG GG-3′, supplemented with the *Pst1* restriction site. The episomally expressed pBAV1K-T5-gfp carrying the kanamycin resistance (Addgene; Cambridge, MA) was digested with the corresponding restriction enzymes. Following ligation, the expression of the *nox* gene will be control by the T5 promoter. Following ligation, *E. coli* DHα was transformed with the pBAV1K-T5-gfp*^nox^*. The pBAV1K-T5-gfp*^nox^* was purified using a plasmid DNA purification kit (Macherey Nagel, Durin, Germany) and transformed into CSP1 treated Δ*nox* WU2. The existence of the *nox* gene in the Δ*nox* WU2*^nox^* was verified by PCR and sequencing. Moreover, *S. pneumoniae* WU2 WT, Δ*nox* WU2*^nox^*, Δ*nox* WU2 and Δ*nox* WU2^empty plasmid^ were grown in THY to OD_600_ 0.2 centrifuged, lysed using urea buffer and NOX protein expression was verified by immunoblotting the complemented Δ*nox* WU2 (Figure S1 in File S1).

### Flow cytometry

Flow cytometry was performed as previously described [25]. PBS used for blocking, staining and washing was supplemented with 2% (v/v) fetal calf serum (FCS) and 0.05% sodium azide. Following blocking, unencapsulated *S. pneumoniae* R6 strain bacteria were incubated with mouse anti-rNOX antiserum or preimmune mouse serum, washed, and stained with FITC-conjugated-F(ab′)_2_-Goat-anti-mouse-IgG+IgM (Jackson ImmunoResearch, West Grove, PA). Flow cytometry was performed using a FACS Calibur flow cytometer (Becton Dickinson, Mountain View, CA) and data were acquired and analyzed using BD CELLQuest™ 3.3 software.

### Transmission electron microscopy

The bacterial pellet from a mid-log culture of unencapsulated *S. pneumoniae* R6 strain bacteria was resuspended in PBS and droplets were placed on Formvar/carbon-coated copper mesh grids (EMS, Hatfield, PA) blocked with 1% BSA (in PBS) followed by incubation with mouse anti-rNOX antiserum or preimmune mouse serum. Bound antibodies were detected using protein A-conjugated gold particles (Jackson Immunoresearch Laboratories, West Grove, PA). Bacteria were examined on a JEM-1230 (JEOL Instrument, Inc., Japan) transmission electron microscope (TEM) operating at 120 kV and photographed at a magnification of ×50,000.

### *S. pneumoniae* adhesion to A549 cells assay

A549 cells (lung adenocarcinoma cells; ATCC, Rockville, MD, USA) retain morphological, biochemical and immunological characteristics resembling type II lung epithelial cells [28]–[30] and have been widely used as a model to study *S. pneumoniae* interaction with human cells [31], [32]. A549 cells were cultured on fibronectin-coated 96-well plates (5×10^5^cells/well) in DMEM (without antibiotics). Wild type WU2, Δ*nox* WU2, Δ*nox* WU2*^nox^*, Δ*nox* WU2^empty plasmid^ (10^7^ CFU; MOI 20∶1) were added to the cells for 1 hr incubation at 37°C. Excess bacteria were removed, and the cells were detached (0.25% trypsin-EDTA) and plated onto blood agar plates for enumeration. This experiment was performed in triplicate and repeated three times. rNOX at concentrations of 0–600 nM was incubated with the cells for 1 hr. To verify that the tag does not affect *S. pneumoniae* adhesion to the A549 cells, the ability of the tagged and the tagged protein to inhibit adhesion to the A549 cell of all the bacterial strains, mentioned in the manuscript, was tested with similar results. However, to prevent redundancy, representative results are included demonstrating untagged rNOX experiments with serotype 3 strain WU2 and its unencapsulated derivative strain 3.8 and HAT-tagged rNOX experiments with serotype 2 strain D39 and its unencapsulated derivative strain R6. Following removal of the excess protein, *S. pneumoniae* (10^7^ CFU/well; MOI 20∶1) were added for 1 hr incubation. Excess bacteria were removed and the cells were released (0.25% trypsin-EDTA) and plated onto blood agar plates for enumeration. The experiment was performed in triplicate and repeated three times.

Inhibition of *S. pneumoniae* adhesion to A549 cells by anti-rNOX antiserum, selected phages or synthetic peptides was performed as previously described [25], [33]. In brief, *S. pneumoniae* were incubated with antiserum, phages or synthetic peptides for 1 hr and then added to the cultured A549 cells for 1 hr incubation. Excess bacteria were removed and the cells were released (0.25% trypsin-EDTA) and plated onto blood agar plates for enumeration. The experiment was performed in triplicates, and repeated three times.

### Inoculation of mice

The effect of ablation of *nox* on the virulence of *S. pneumoniae* was tested using BALB/c OlaHsd mice (BALB/c; Harlan Laboratories, Israel) described as being relatively resistant to *S. pneumoniae* infection. In an attempt to increase signal-to-noise responses, similar experiments were performed using CBA/CaHN-*Btk^xid^*/J mice (Jackson Laboratories, Bar Harbor, ME), a strain highly susceptible to *S. pneumoniae* infection [22], [34]. This mouse strain has a mutation in the Bruton kinase gene, which renders them unable to launch an antibody response to thymus-independent type II antigens such as the capsular polysaccharide, although they do produce normal amounts of antibodies in response to some protein antigens [35].

Eight-week-old BALB/c mice (3 in each group) were inoculated intranasally (IN) with a sublethal dose (5×10^7^ CFU) of WT WU2, Δ*nox* WU2, Δ*nox* WU2*^nox^* and Δ*nox* WU2^empty plasmid^. Forty-eight hours later, the mice were euthanized, and the nasopharynx and lung were removed, homogenized and plated onto blood agar plates for enumeration. BALB/c mice were inoculated IN with a lethal dose (1×10^8^ CFU) of WT WU2 (n = 12) or Δ*nox* WU2 (n = 12), or intraperitoneally (IP) with either WT WU2 (n = 10; 64 CFU) or Δ*nox*WU2 strains (n = 10; 70 CFU), and survival was monitored daily. We also inoculated 7- to 9- week-old CBA/N*xid* mice IN (5×10^5^ CFU) with either WT WU2 (n = 10) or Δ*nox* WU2 (n = 10), or IP with either WT WU2 (n = 10; 64 CFU) or Δ*nox* WU2 (n = 10, 70 CFU). The groups were matched for age. Survival was monitored daily. These experiments were repeated 3 times on three different occasions.

### Identification of rNOX binding phages

To identify putative rNOX-binding sequences, a combinatorial peptide library expressed in a filamentous phage *fth1* was screened as previously described [36]. The 12-mer cysteine-constrained random peptide library was constructed in the PVIII proteins of a mosaic fd phage. Selection and characterization of phages were accomplished via Tetracycline^R^ bacterial colonies. Following incubation of the phages on HAT-rNOX adsorbed culture dishes, unbound phages were washed out and the bound phages were eluted at pH 2.2. Nine of the rNOX-binding phages were tested for their ability to interfere in bacterial adhesion to cultured A549 cells.

### Enrichment of *S. pneumoniae* WU2 strain cell-wall non-lectin (CW-NL) proteins

Bacteria were treated with mutanolysin to release cell-wall proteins [37]. The cell-wall proteins were separated on fetuin-agarose columns as described by Sela *et al* [38]. The flow-through fraction was considered as the non-lectin-binding (NL) fraction. Protein content was quantified by BCA™ Protein Assay Kit (Pierce Biotechnologies, Inc. Rockford IL).

### Active immunization of mice with rNOX

BALB/c mice were immunized subcutaneously with either with PBS, or 5 or 25 µg rNOX or 25 µg of NL fractions (three mice in each group) emulsified with CFA and boosted (days 14 and 28) with IFA. Mice were challenged intranasally (IN) on day 42 with a sublethal dose of *S. pneumoniae* WU2 strain (5×10^7^). Mice were eutinized 48 h later; the nasopharynx and right lobe lung were excised, homogenized and plated onto blood agar plates for bacterial enumeration.

BALB/c mice were immunized subcutaneously with PBS (n = 14), 25 µg of rNOX (n = 12) or 25 µg of NL fractions (n = 8) emulsified with CFA and boosted (days 14 and 28) with IFA. Two weeks after last immunization, mice were inoculated IP with WU2 strain (250 CFU/50 µl PBS) and survival was monitored daily.

### Ex vivo neutralization of *S. pneumoniae* with anti-rNOX antisera

A lethal dose (250 or 500 CFU) of *S. pneumoniae* WU2 strain was incubated at 37°C for 1 hour with diluted rabbit pre-immune, anti-rNOX, or anti-NL sera, and subsequently inoculated IP to BALB/c. Survival was monitored daily.

### Bioinformatics analysis

The nucleotide and amino acid sequences of NOX were analyzed for homology to human or *S. pneumoniae* sequences using the NCBI database. The nucleotide sequences of the insert peptide in rNOX-binding phages was converted into an amino acid sequence and compared to the entire human genome in NCBI and UCSC databases. A random peptide sequence was formulated by bioinformatics (LPADWATTLMVCSSK), synthesized and used as a negative control.

### Statistical analysis

The Spearman correlation analysis was used to evaluate the dose dependency inhibition of *S. pneumoniae* adhesion to A549 cells. Significance of inhibition was determined by two tailed Student's t-test. Inhibition of bacterial colonization was established using the Student's t-test. Analysis of survival was carried out using the Log rank (Mantel Cox) test.

## Results

### Characteristics of NOX

#### Age-dependent antigenicity

**Figure 1:**
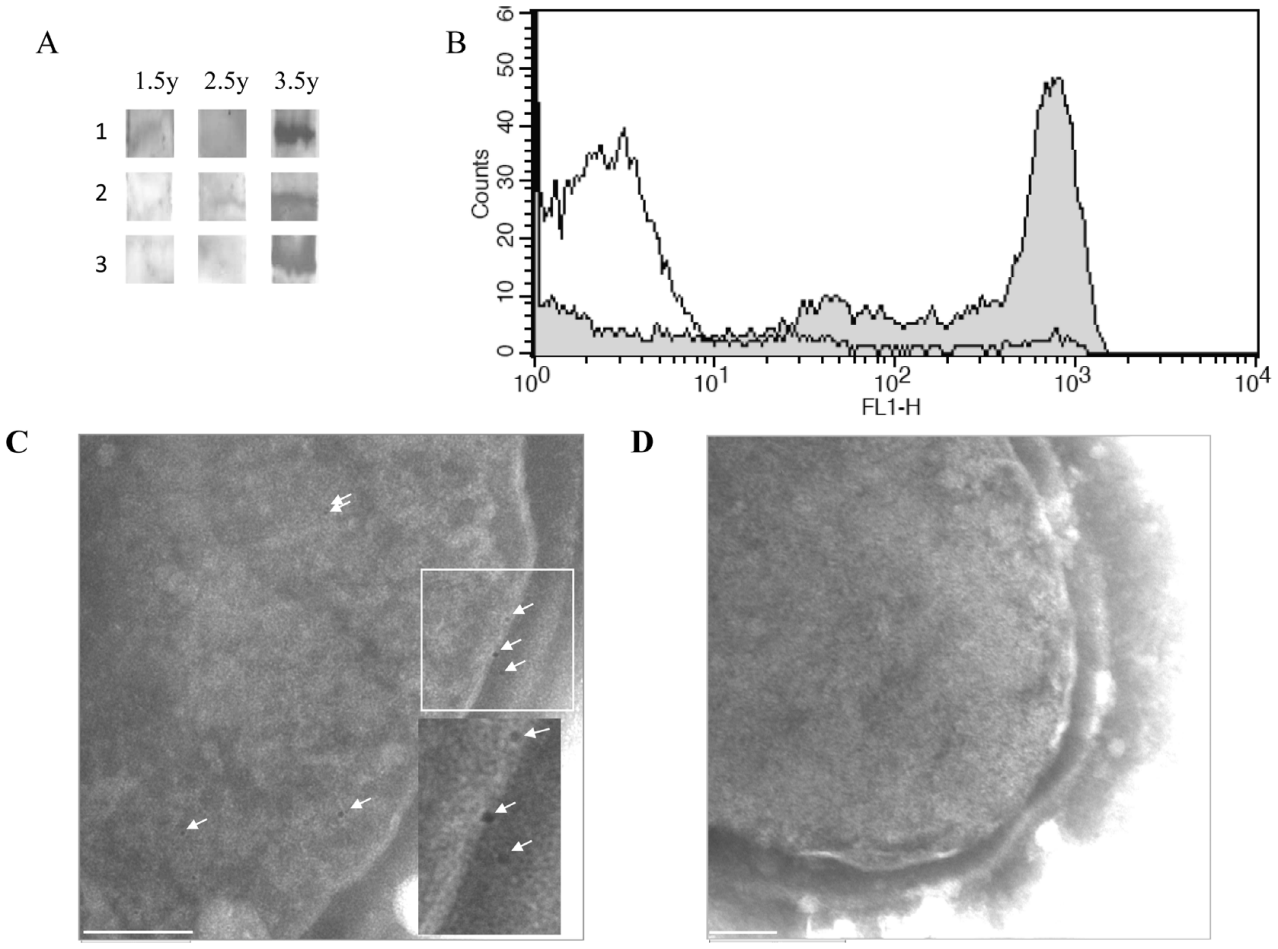
Characteristics of NOX. A. **Age-dependent antigenicity.** Immunoblotting of SDS-PAGE-separated rNOX-HAT fusion protein with sera obtained longitudinally from three infants at 18, 30 and 42 months of age. B. ***Surface expression of NOX.*** FACS histogram overlay shows the unencapsulated R6 strain stained with mouse anti-rNOX antiserum (filled) or with preimmune mouse serum (unfilled). C and D. ***Surface expression of NOX.*** Wild type R6 (C) and Δ*nox*R6 (D) strains were probed with rabbit anti-rNOX antiserum. Bound antibodies were labeled with protein A-conjugated gold particles (arrowheads) and visualized by TEM. Bar, 100 nm.

An immunoblot analysis of rNOX using sera drawn longitudinally from three healthy infants showed that sera at 42 months of age demonstrated augmented recognition of rNOX in comparison to sera obtained from the same children at 18 or 30 months of age (Figure 1A). In addition we tested sera obtained from different children at 7, 12, 24 and 38 months of age and 4 sera from random mothers. While at 7 months of age rNOX was recognized strongly only in 1 out the 4 children, at 12 months all 4 children's sera had anti-rNOX antibodies, albeit the recognition of rNOX was to a low extent. At 24 months of age 2 out of the 4 had antibodies to rNOX and at 38 months of age rNOX was recognized strongly with the children's sera to same extent as the recognition of rNOX with sera obtained from the random mothers (Figure S2 in File S1).

#### Surface expression of NOX

We have previously found NOX to be localized to the *S. pneumoniae* cell-wall [20]. *S. pneumoniae* strain R6 bacteria were stained with murine anti rNOX antiserum and flow cytometry analysis revealed the cell-wall localization of NOX (Figure 1B). The flow cytometry analysis further strengthened the notion that NOX is localized at the surface of the cells. This notion received further fortification from the TEM results, which showed that NOX could be detected on the surface of wild type but not on the surface of the NOX-null R6 strain (representative images shown in Figure 1C).

#### Reduced adhesion and virulence of Δ*nox S. pneumoniae*

**Figure 2:**
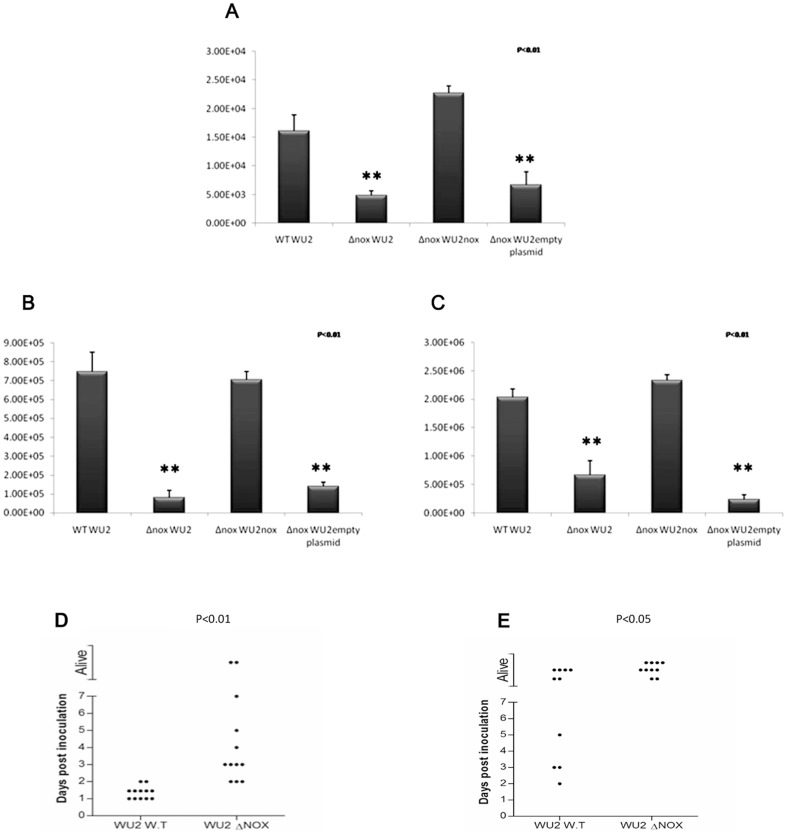
Reduced adhesion and virulence of Δ*nox S. pneumoniae*. A. Wild type (WT), Δ*nox* mutants, Δ*nox* mutants or Δ*nox* mutants^empty plasmid^ bacteria (10^7^ CFU; MOI 20∶1) were added to the cells for 1 hr incubation at 37°C. Excess bacteria were removed and the cells were released (0.25% trypsin-EDTA) and plated onto blood agar plates for enumeration. The experiment was performed in triplicate, and repeated three times. B and C. BALB/c mice were inoculated IN with either WTWU2, Δ*nox* WU2 or Δ*nox* WU2*^nox^* or Δ*nox* WU2^empty plasmid^ (5×10^7^ CFU). Forty eight hours following inoculation the nasopharynx (B) and lungs (C) were excised, homogenized and plated onto blood agar plates for enumeration. D. BALB/c mice were inoculated IN with a lethal dose (1×10^8^ CFU) of WT WU2 (n = 12) or Δ*nox* WU2 (n = 12). Survival was monitored daily; each point on the scatter plots represents an individual mouse. E. BALB/c mice were inoculated intraperitoneally (IP) with either WT WU2 (n = 10; 64 CFU) or Δ*nox*WU2 strains (n = 10; 70 CFU). Survival was monitored daily; each point on the scatter plots represents an individual mouse.

In order to explore the putative involvement of cell-wall localized NOX in bacterial-host interaction, we compared the extent of adhesion of the parental WU2 and its NOX-null (Δ*nox* WU2) mutant strains to A549 lung epithelial adenocarcinoma cells *in vitro*. To verify that the reduced adhesion of Δ*nox*WU2 bacteria to A549 cells results from ablation of *nox* expression only, we complemented the Δ*nox* WU2 with a *nox* gene inserted into the pBAV1K-T5-gfp plasmid (pBAV1K-T5-gfp*^nox^*). No significant differences could be found in the number of bacteria recovered from A549 cells infected with WT WU2 and the complemented Δ*nox* WU2*^nox^* bacteria. However, the number of bacteria recovered from A549 infected with the Δ*nox* WU2 and Δ*nox* WU2^empty plasmid^ bacteria was significantly reduced (Figure 2A; *p*<0.05).

To test the effect of *nox* on *S. pneumoniae* virulence, BALB/c mice were inoculated intranasally with the WT WU2 strain, Δ*nox* WU2, Δ*nox* WU2*^nox^* or Δ*nox* WU2^empty plasmid^. Mice inoculated IN with WT WU2 and the complemented Δ*nox* WU2*^nox^* demonstrated similar virulence, as determined by the bacterial load in the nasopharynx and the lungs 48 hours following inoculation (Figures 2B and 2C, respectively). However, the number of bacteria recovered from the BALB/c mice inoculated IN with Δ*nox* WU2 and the Δ*nox* WU2^empty plasmid^ demonstrated a significantly reduced number of bacteria recovered from the nasopharynx and the lungs, suggesting that the *nox* null mutants have reduced virulence (Figure 2B and 2C; *p*<0.01 ).

Survival rate was significantly higher in BALB/c mice inoculated IN with Δ*nox*WU2 compared to the WT WU2 (Figure 2D). Similar results were obtained in CBA/N*xid* mice inoculated IN with WT WU2 or Δ*nox* WU2 strains (Figure S3A in File S1).

To further investigate whether the attenuated virulence is independent of NOX enzymatic activity, bacteria were administrated to an anaerobic niche, the peritoneum [39]. The survival rates of mice challenged with Δ*nox* WU2 were higher than those challenged with the WT parental strain both in BALB/c (Figure 2F; *p*<0.05) and CBA/N*xid* mice (Figure S3B in File S1).

#### *S. pneumoniae* adhesion to A549 is mediated by NOX

**Figure 3:**
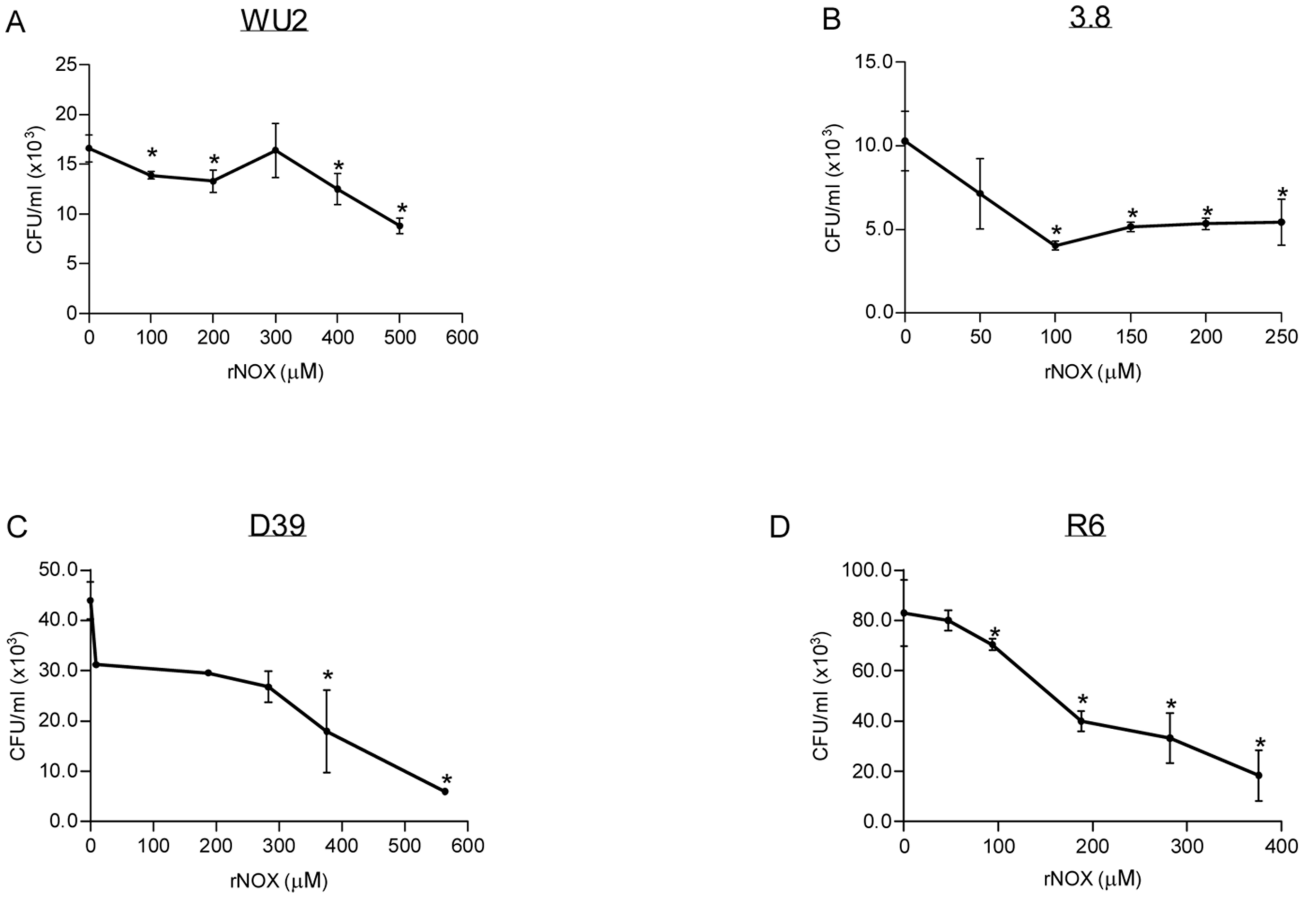
*S. pneumoniae* adhesion to A549 is inhibited by rNOX. rNOX protein, at the indicated concentrations, was incubated with cultured A549 cells for 1 hr. Following removal of excess protein, *S. pneumoniae* were added and incubated for an additional 1 hr with the cultured cells. Excess bacteria were removed, and the cells were detached and plated onto blood agar plates for bacterial enumeration: A. WU2 strain (t-test p<0.05; Spearman r = −828 p<0.05) B. 3.8DW strain (t-test p<0.05, Spearman r = −428, p = 0.414); C. D39 strain (t-test p<0.05, Spearman r = −952, p<0.01); and D. R6 strain (t-test p<0.05, Spearman r = −0.862 p<0.05).

NOX involvement in *S. pneumoniae* -host cells interaction was further studied by analyzing the ability of rNOX to compete with *S. pneumoniae* adhesion to A549 lung derived adenocarcinoma cell line. Untagged rNOX significantly inhibited the adhesion to A549 cells in a dose-dependent manner of encapsulated serotype 3 strain WU2 (t-test p<0.05; Spearman r = −828 p<0.05) and its unencapsulated derivative, 3.8DW (t-test p<0.05, Spearman r = −428, p = 0.414). Tagged rNOX significantly inhibited the adhesion of serotype 2 D39 strain (t-test p<0.05, Spearman r = −952, p<0.01) and its unencapsulated derivative R6 (t-test p<0.05, Spearman r = −0.862 p<0.05) strains. The half maximal inhibitory concentration (IC_50_) of rNOX was determined, demonstrating IC_50_ = 350 µM for the WU2 strain, IC_50_ = 50 µM for the 3.8DW strain, IC_50_ = 379.9 µM for the D39 strain and IC_50_ = 274.3 µM for the R6 strain (Figure 3A–D).

**Figure 4:**
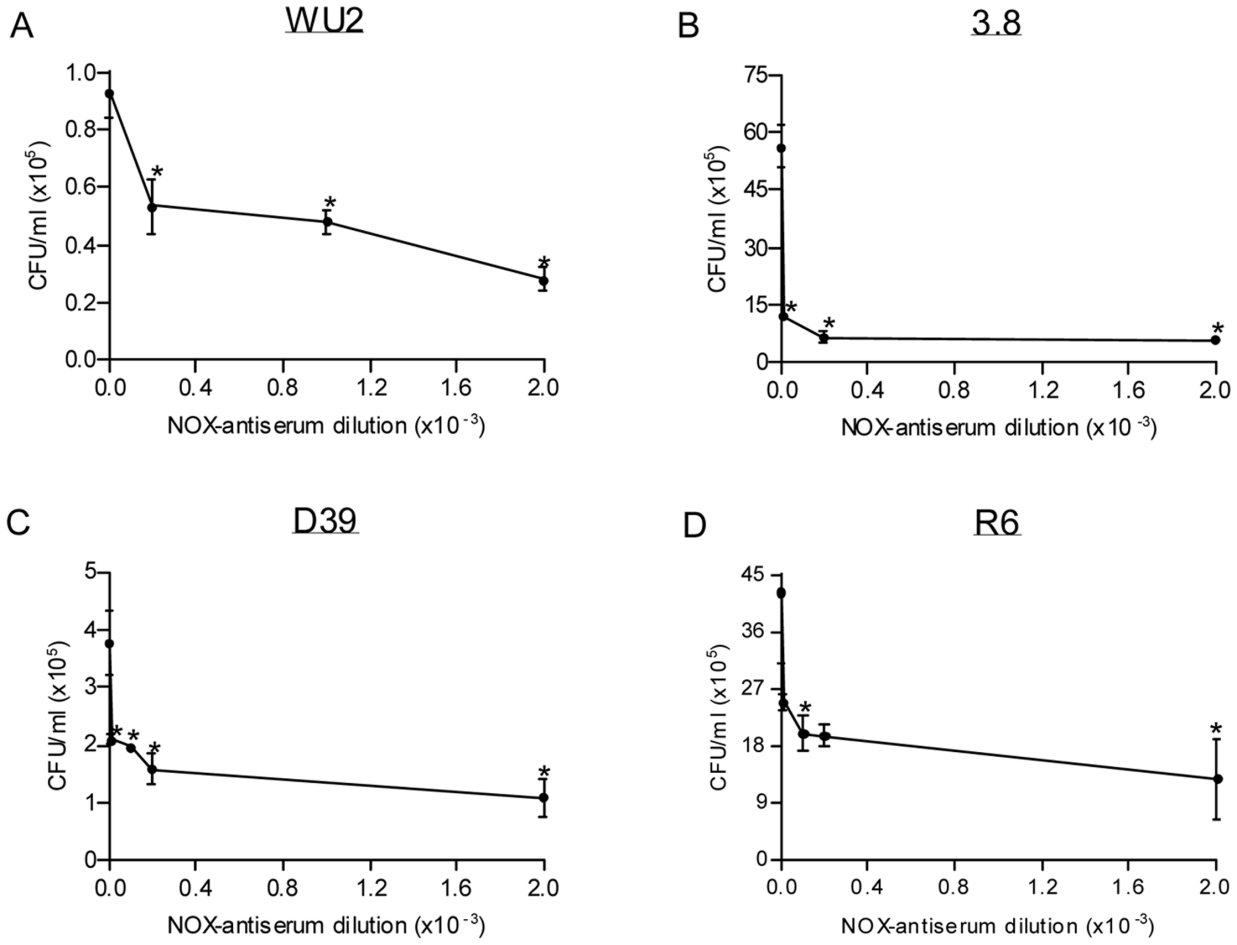
Inhibition of *S. pneumoniae* adhesion to A549 cells by anti-rNOX antiserum. *S. pneumoniae* strains (10^6^ CFU): A. WU2 strain; B. 3.8DW strain; C. D39 strain; and D. R6 strain were incubated with A549 cells following 30 min incubation with the indicated dilutions of rabbit anti-rNOX antiserum. Excess bacteria were removed, and the cells were detached and plated onto blood agar plates for bacterial enumeration. A significant dose-dependent inhibition of bacterial adhesion was observed using Spearman regression analysis (A, C, D) or *t-test* analysis (B) in comparison to the untreated bacteria.

The ability of rabbit anti-rNOX antiserum to neutralize *S. pneumoniae* surface- expressed NOX and consequently inhibit the bacteria adhesion to A549 cells was also tested. Preincubation of A549 cells with rabbit anti rNOX antiserum led to impaired bacteria-target cell interaction in a dose-dependent manner. Rabbit anti-rNOX antiserum was found to maximally inhibit the adhesion of 74% of the WU2 strain (t-test p<0.05, r = −0.855), 88% of the 3.8DW strain (t-test p<0.05), 80% of D39 strain (t-test p<0.05, r = −0.568) and 70% of R6 strain (t-test p<0.05, r = −0.613; Figure 4A–D).

#### Identification of rNOX-binding sequences

A combinatorial peptide library expressed in a filamentous phage was screened in order to identify rNOX-binding ligands using the HAT-tagged rNOX. Nine rNOX-binding phages were recovered and tested for their ability to interfere with bacterial adhesion to cultured A549 cells. Five phages were found to significantly inhibit *S. pneumoniae* 3.8DW strain adhesion to A549 cells (t-test, p<0.05, data not shown). The peptide inserts in the five inhibitory phages were sequenced and aligned against the human genome. The insert peptide in one phage (WQNXRPKPTYAQ) was similar to amino acids 7–19 in the cell adhesion molecule contactin-4. The insert peptide in a second phage demonstrated a five amino acid similarity to a sequence within Chondroitin 4 sulfotransferase (ASARE); furthermore, amino acid sequence ASAR showed similarity to a sequence within Collagen XI. The identified peptide sequence in a third phage (CQEXSPG) demonstrated similarity to two distinct areas in the extra-cellular matrix protein laminin alpha-5 (amino acids 661–667 and amino acids 1842–1848). The insert peptides in the fourth phage (LCCEKH) demonstrated similarity to sequences within the cell membrane protein Disintegrin (Table 1). The insert peptide in the fifth phage (VGGSRDNRGGAHDDRNKSRPNRXR) demonstrated homology to glutamate receptor interacting protein.

**Table 1:** Homologous sequences in human proteins to the insert peptides in the inhibitory phages.

**Phage number 1**	QNXRPKPTYAQ
Contactin 4 (Q8IWV2 UniProt) C4P	^7^WECKANGRPKPTY^19^
**Phage number 2**	VHXAXKSASARE
Chondroitin 4 sulfotransferase (Q9NRB3 UniProt) CSP	^279^ANHTSLPASARE^290^
Collagen XI	^1704^ASAR^1707^
**Phage number 3**	GCQEXSPGT
Laminin (015230 UniProt) A5P	^0661^CQECSPGF^668^
	^1842^CQECSPGF^1849^
**Phage number 4**	IVSTXNLCCFKHP
Disintegrin/Metalloprotease (Q9BZ11 UniProt)	^442^LCCFAHN^447^
**Phage number 5**	VGGSRDNR-GGAHDDRNKSRPNRXR
glutamate receptor interacting protein (Q9Y3R0 UniProt)	^168^GGAHDDRNKSRP^179^

#### NOX target peptide inhibits NOX adhesion to cultured A549 cells

**Figure 5:**
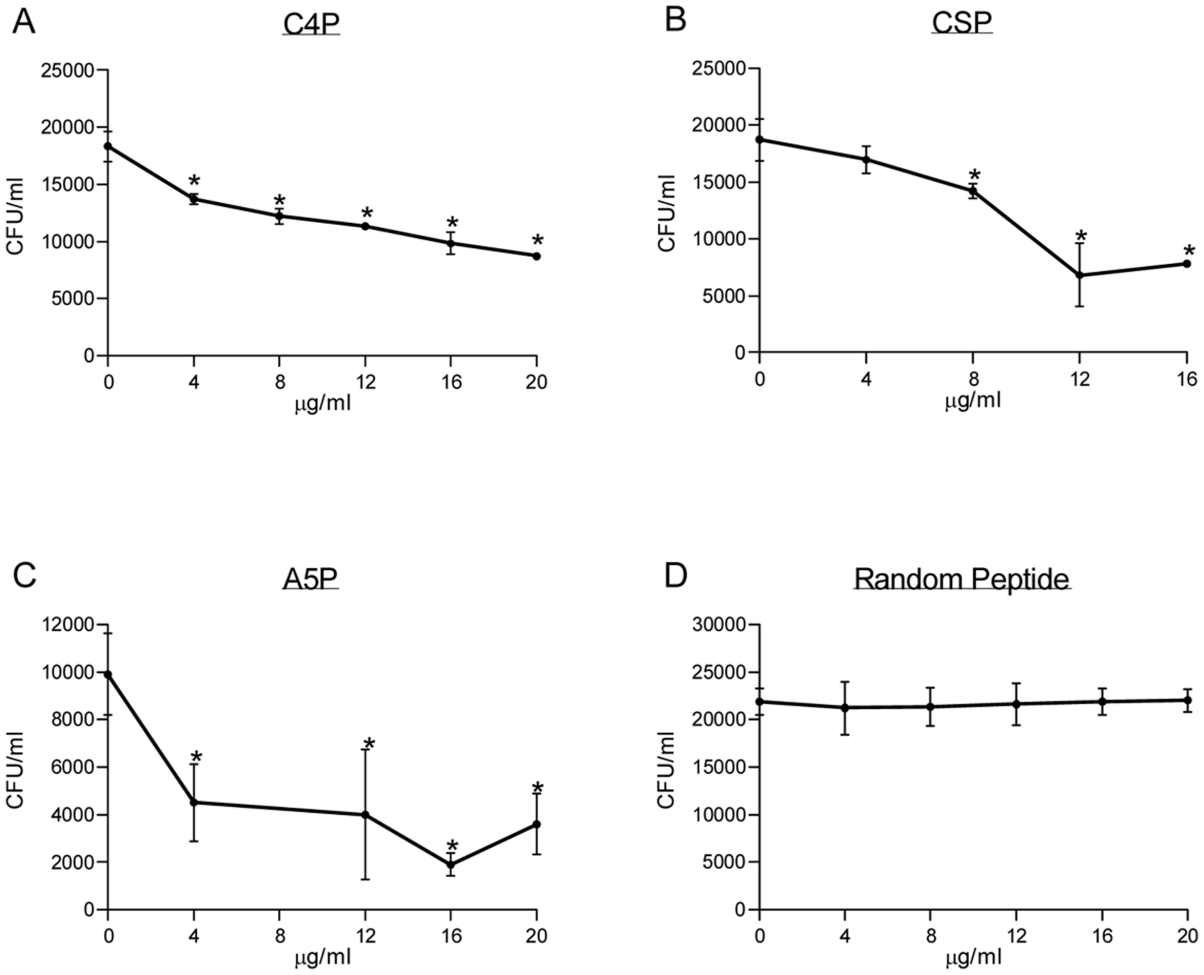
Inhibition of *S. pneumoniae* adhesion to A549 cells by NOX target peptides. *S. pneumoniae* serotype 3 strain WU2 was incubated for 60 min. with the corresponding peptide at the denoted concentration. The bacteria/peptide suspension was added to the cultured A549 cells for 60 minutes incubation. Following incubation, excess peptide/bacteria suspension was removed, and the cells were detached and plated onto blood agar plates for bacterial enumeration. A significant dose-dependent inhibition of bacterial adhesion was observed in Spearman regression analysis. A. Contactin 4 derived peptide (C4P, r = −0.889, t' test p<0.05), B. Chondroitin 4 Sulfotransferase derived peptide (CSP, r = −0.705, t' test p<0.05). C. Alpha 5 Laminin (A5P, r = −0.643, t' test p>0.05).

Based on the known biological function of the identified human proteins we chose to further study three out of the five peptides. We synthesized peptides derived from the human protein homologous to the insert peptides in the inhibitory phages. Peptides derived from contactin 4 (C4P), Chondroitin 4 Sulfotransferase (CSP) and alpha 5 laminin (A5P) were tested for their ability to inhibit bacterial adhesion of *S. pneumoniae* to A549 cells. C4P significantly inhibited *S. pneumoniae* strain WU2 adhesion to A549 epithelial cells in a dose dependent manner (Figure 5A; t-test p<0.05 Spearman r = −0.889, p<0.05). Maximal inhibition observed was 40.2% with 12.9 µM C4P. CSP significantly inhibited *S. pneumoniae* strain WU2 adhesion to A549 epithelial cells in a dose dependent manner (Figure 5B, t-test p<0.05; Spearman r = −0.705, p<0.05). Maximal inhibition observed was 63.64%, obtained with 9.6 µM CSP. A5P significantly inhibited *S. pneumoniae* strain WU2 adhesion to A549 epithelial cells in a dose dependent manner, (Figure 5C, t-test p<0.05; Spearman r = −0.643, p<0.05. More than 90% adhesion inhibition of WU2 strain to A549 cells could be observed starting at concentration of 5.85 µM. A negative control random peptide selected did not inhibit bacterial adhesion to the cultured A549 cells (Figure 5D).

Using a dot blot analysis A5P and C4P bound the untagged rNOX (Figure S4 in File S1) as well as the tagged rNOX (Data not shown) significantly as detected by rabbit anti-rNOX antibodies. However, the rNOX proteins did not detect the CSP. It may be that the affinity of the rNOX protein currently in use for this peptide is reduced in comparison to its affinity to the native protein as it inhibits bacterial adhesion to the A549 cells. Alternatively, a more unlikely possibility is that it is a result of binding to another contaminating unknown adhesin.

#### Vaccine potential of rNOX

Bioinformatic analysis demonstrated that NOX is highly conserved (99–100%) among 38 different *S. pneumoniae* strains sequences (UniProt BLASTP; using build uniprotkb (Protein) generated for BLAST on Feb 7, 2011). In order to consider NOX as a vaccine candidate, homology to human proteins was analyzed. The human protein with the highest general identity to NOX was found to be Apoptosis-inducing factor 3 isofirm 1 (NP_653305) demonstrating 25% general identity, where no clusters exceeding three amino acids in a row were found. These data combined with the cell-wall localization of NOX, age-dependent antigenicity of NOX and the putative function of NOX as an adhesin encouraged testing NOX for its vaccine efficacy.

**Figure 6:**
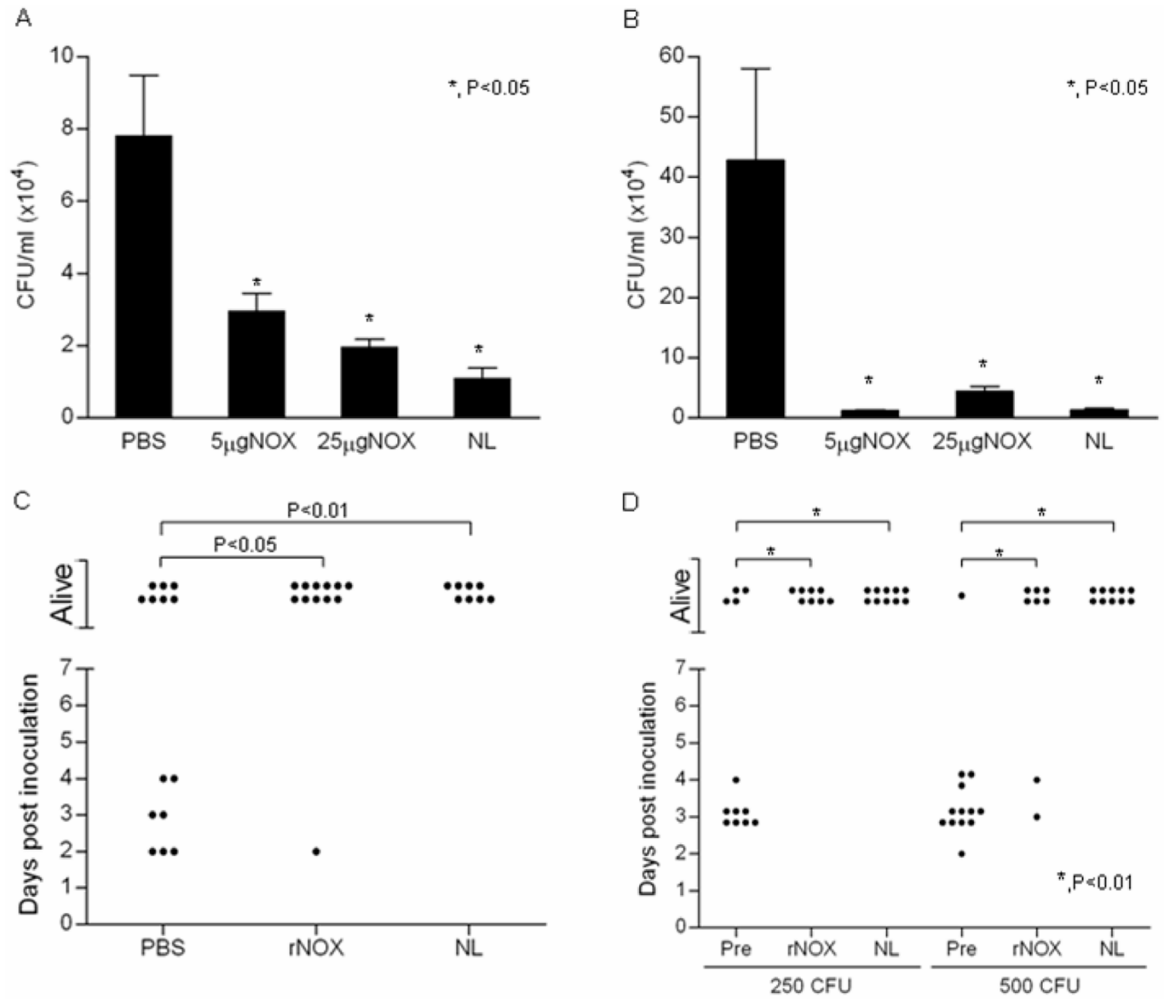
rNOX elicits a protective immune response against *S. pneumoniae*. *A and B*, Seven-week-old BALB/c mice were immunized SC with 5 and 25 µg rNOX. Negative control immunization included adjuvant-only and positive control included the NL fraction. Mice were challenged 14 days after last immunization with 5×10^7^ CFU WU2 strain. Forty-eight hours after inoculation, mice were euthanized and the nasopharynx and lungs (A and B, respectively) were harvested, homogenized and plated onto blood agar plates for bacterial enumeration. C. Mice were challenged IP (250 CFU WU2 strain) 10 days after last immunization. Survival was monitored daily; each point on the scatter plot represents an individual mouse. D. The indicated amount of WU2 strain were incubated with 1∶5 dilution of pre-immune rabbit serum or rabbit anti-rNOX antiserum or rabbit anti-NL antiserum, and then inoculated IP to BALB/c mice. Survival was monitored daily; each point on the scatter plots represents an individual mouse.

BALB/c mice were subcutaneously immunized with rNOX and challenged intranasally (IN) with WU2 strain one week after the last immunization. Forty eight hours following inoculation, bacterial colonization in the nasopharynx (Figure 6A) and lungs (Figure 6B) were significantly reduced in the rNOX-immunized mice in comparison to the adjuvant only immunized mice (p<0.05).

Furthermore, rNOX immunized BALB/c mice were significantlly protected from an intraperitoneal (IP) inoculation with WU2 strain (Figure 6C, p<0.01;).

In order to further establish the putative function of NOX as an adhesin, we tested the ability of heat inactivated NOX-specific antiserum to neutralize *S. pneumoniae* virulence. WU2 strain bacteria (250 or 500 CFU) were incubated with rabbit pre-immune serum, rabbit anti-rNOX antiserum, or rabbit anti-NL antiserum (positive control). The bacteria-serum mixtures were then inoculated IP to BALB/c mice. Signficantly reduced mortality was observed in mice inoculated with bacteria pretreated with rabbit anti-rNOX or rabbit anti-NL antisera in comparison to mice inoculated with bacteria treated with pre-immune serum (Figure 6D, p<0.01).

## Discussion

Genome analysis of *S. pneumoniae*, an anaerobic aero-tolerant bacterium, reveals the absence of most of the genes involved in aerobic respiration, tricarboxylic acid cycle and the electron transport chain [40]–[42]. Nevertheless, pneumococci have oxidases, such as pyruvate oxidase and NADH oxidase (NOX) capable of reducing molecular oxygen [43]. Previously published data attributed the importance of NOX in *S. pneumoniae* pathogenesis to its oxidase activity demonstrating reduced growth rates of Δ*nox* mutants under aerobic but not anaerobic conditions [19], [43]. The cell–wall localization of NOX, previously described by us [20], has been further strengthened by our current studies utilizing flow cytometry and TEM. The cell-wall localization of NOX suggests the existence of a function other than its oxygen reducing enzymatic activity. Surface localization and a moonlighting role in pathogenesis for enzyme proteins lacking a known export signal has been demonstrated, among others, in *S. agalactiae*, *S. pyogenes* and *S. pneumoniae* [44].

A deletion mutation of *nox* reduces bacterial adhesion to A549 cells and diminishes pneumococcal virulence in the intranasal as well as in the intraperitoneal challenge models in mice. The effect on bacterial virulence in aerobic niches can be a consequence of the NOX oxygen reducing function. However, the peritoneum is considered an anaerobic environment [39] thus the reduced virulence of Δ*nox* WU2 following intraperitoneal challenge may result from loss of a function other than the enzymatic activity of NOX. Our observation that Δ*nox* WU2 bacteria demonstrate reduced adhesion capacity to the A549 cells *in vitro* further suggested that NOX may function as an adhesin. Complementation of the mutant Δ*nox* WU2 with the *nox* gene in a shuttle vector, but not the empty vector, restored bacterial adhesion to A549 cells *in vitro* and virulence *in vivo*. The reduced virulence *in vivo* could be demonstrated by the reduced bacterial load in the nasopharynx and lungs of mice inoculated intranasally with the bacteria lacking *nox* and the increased survival rates of mice inoculated either IN and IP, suggesting that no other genes were affected by the ablation of *nox* in the bacteria.

*S. pneumoniae* pathogenesis involves bacterial spread from the nasopharynx either to the middle ear or to the lung or in some cases to the blood stream and the brain. Thus, this bacterium has developed multiple adhesins to facilitate its retention in different tissues following dissemination. The process of adhesion is accompanied by shedding of the bacterial capsule and exposure of its cell-wall- and membrane-embedded adhesins [45]. Shedding of the bacterial capsule during adhesion may explain the ability of rNOX and anti-rNOX antibodies to interfere with encapsulated bacterial adhesion to target mammalian cells either by competing for the receptor or neutralizing the bacteria, respectively. The mutant Δ*nox* WU2 exhibited reduced adhesion, however this was not as strong as the adhesion inhibition induced by rNOX and anti-rNOX antibodies. This may be explained by rNOX binding to target molecules shared with other adhesins or alterations in the mutant that compensated for NOX deletion

To identify rNOX-binding sequences, we screened a combinatorial peptide library expressed in a filamentous phage [36]. Some of the rNOX-binding phages inhibited bacterial adhesion to A549 cells. The insert peptides in one of these phages aligned to human Contactin 4, a cell adhesion molecule. Cell adhesion molecules in general are known targets for many pathogens [46], [47]. The insert peptide in three other phages aligned to two extracellular matrix (ECM) proteins: laminin 5 alpha and collagen XI. Pathogen-mucosal cell interactions promote epithelial and endothelial cells apoptosis, exposing components of the extracellular matrix which were also demonstrated to mediate various pathogen interactions with the host [48]. In the case of ECM proteins it can be assumed that they will bridge between the pathogen and the host cellular receptor [12]. An insert peptide in another phage aligned to Chondroitin 4 sulfotransferase. This enzyme catalyzes the transfer of sulfate to Chondroitin [49]. It was found to be secreted from culture chondrocytes and may thus reside in the ECM [48]. Another cell membrane protein which aligns to an insert peptide in an additional phage is the disintegrin/metaloproteinase. It is expressed in all tissues except the liver. It is highly expressed in the lungs (Q9BZ11 UniProt) [50]. The fifth insert peptide aligned to glutamate receptor interacting protein 1 (GRIP1), a member of the glutamate receptor interacting protein family. This is a scaffold protein that binds to and mediates the trafficking and membrane organization of a number of trans-membrane proteins (Q9Y3R0 UniProt).

Based on the known biological function of the identified human proteins, we chose to further study three out of the five target derived peptides. Peptides synthesized from Contactin 4, Chondroitin 4 sulfotransferase and laminin 5 alpha were found to interfere with *S. pneumoniae* adhesion to cultured A549 lung derived epithelial cells. These results further suggest that these identified target proteins may be involved in NOX interaction with the host.

The shortcomings of the current polysaccharide vaccines encourage exploring additional strategies to identify serotype-independent protein-based vaccines focusing on immunogenic surface proteins [51], [52]. We have previously described the identification of a group of *S.pneumoniae* surface proteins with age dependent antigenicity. The increase in the age dependent antigenenicity correlates negatively with morbidity suggesting that this increase may signify the development of natural immunity to this bacterium. Indeed several proteins from this group tested to date were found to elicit a partially protective immune response in mice models [25], [33], [53].

The surface localization of NOX, its age dependent antigenicity, its possible involvement in adhesion to the host, its low homology to human sequences and high conservation between *S. pneumoniae* stains encouraged analysing its vaccine potential. Immunization of mice with rNOX elicited a protective immune response to intranasal as well as to intraperitoneal challenges. The reduced colonization observed in the nasopharynx and in the lungs of immunized mice following intranasal challenge could result from the neutralization of NOX enzymatic activity in these aerated tissues. The protection gained against intraperitoneal challenge following immunization with rNOX suggests that the protective mechanism can not be attributed to the inhibition of NOX's enzymatic acitivity but rather to its role as an adhesin. This is notion is further strengthend by the ability of anti-rNOX antisera to neutralize *S. pneumoniae ex-vivo*, prior to the intraperitoneal challenge. The ability of rNOX and the target derived peptides to interfere in bacterial adhesion to A549 cells suggests that NOX is involved in adhesion to these type II adenocarcinoma cells. There are no indications that these cells carry FcγRs, suggesting that *in vitro*, the major activity of the antibodies was to interfere in bacterial adhesion to the cells. It should be noted that *in vitro*, the extent of antiprotein antibody binding to encapsulated bacteria is much lower than the observed binding of these antibodies to the cell-wall proteins on unencapsulated bacteria [25]. Thus, the initial activity of these antibodies would be upon the capsule shedding during the initial adhesion of the bacteria to their target cells [45]. However, only few bacteria in the population shed their capsules, as the shedding occurs only on the first bacterium in a chain that binds to a target cell, further suggesting that under this condition inhibition of adhesion is the major function of the antibodies.

In *S. pneumoniae*, anti-capsular polysaccharide antibody involvement in opsonophagocytosis is widely described [54]. However, opsonization with antibodies to cell-wall protein is not easy to demonstrate, thus, few manuscript have been published on this subject [55]. Moreover, the surface exoglycosidases from *Streptococcus pneumoniae*, NanA, BgaA, and StrH, promote resistance to opsonophagocytic killing by human neutrophils [56]. However, *in vivo*, one cannot exclude the possibility that following exposure of the surface protein after capsule shedding the antibodies may participate in an opsonic phagocytosis.

Several strategies have been used to identify pneumococcal serorotype independent protein-based vaccines. Some of those focused on surface immunogenic proteins such as PspA [57], PhtD [58] and PcsB [59]. Other strategies included the identification of *S. pneumoniae* surface proteins involved in bacterial adhesion and invasion of the host [7], [9], [12], [13], [60], [61].

The previously described oxygen reducing activity of NOX together with our current findings suggest that NOX is an important virulence factor. Currently we have demonstrated that NOX may moonlight as an adhesin in the pneumococcal cell-wall. We have then identified putative target molecules and that peptides derived from these molecules could function as “decoy” molecules. The identification of these peptides may lead to development of novel therapeutic molecules as has been previously described for other bacterial and viral systems [62]. Moreover, rNOX protein elicits protective immune response and thus may be considered a candidate for future protein based vaccine.

## Supporting Information

File S1 **Supporting information Figures.** *Figure S1: Age dependent antigenicity to NOX in infants and children*. In attempt to analyze the antibody levels against NOX in children at different ages, rNOX (10 ng) was separated on SDS PAGE, transferred to nitrocellulose membrane. The lanes on the nitrocellulos were cut and probed with sera obtained from different children at 7, 12, 24, and 38 months of age and 4 sera were obtained from random mothers. Sera were diluted 1∶20, the incubation was for the same duration and all the strips were developed together for 20 seconds. The human sera were detected with Goat anti-human IgG-HRP antibody. *Figure S2: Production of null mutant bacteria and its supplementation.* The transformation procedure was performed by homologous recombination as previously described [28]. The genomic DNA of the wild type R6, Δ*nox* R6, WU2 and Δ*nox* WU2 were amplified with the 3′ downwing (IM1) and 5′ upwing (IM4) primers (A) or with primers for erythromycin (B). C and D. Cytoplasmic and cell-wall fractions from R6 WT, Δ*nox* R6 (C), WU2 WT and Δ*nox* WU2 (D) were immunoblotted and probed with rabbit anti-rNOX antibodies. CW: Cell-wall fraction, Cyt: cytoplasmic fraction E. *nox* gene was amplified with primers for *nox* from: WT WU2 (lane 1), Δ*nox* WU2*^nox^* (lane 2) lane 3 Δ*nox* WU2^empty plasmid^ (lane 3) and Δ*nox* WU2 (lane 4). F. *S. pneumoniae* WU2 WT, Δ*nox* WU2*^nox^*, Δ*nox* WU2 and Δ*nox* WU2^empty plasmid^ were lysed and and NOX expression or lack of its expression was verified with rabbit anti NOX antibodies. *Figure S3: Reduced virulence of NOX null mutant bacteria.* A. Seven to 9 week old CBA/N*xid* mice were inoculated IN (5×10^5^ CFU) with either WT WU2 (n = 10) or Δ*nox* WU2 (n = 10) B. Seven to 9 week old CBA/N*xid* mice were inoculated IP with either WT WU2 (n = 10; 64 CFU) or Δ*nox* WU2 (n = 10, 70 CFU). The groups were matched for age. Survival was monitored daily; each point on the scatter plots represents an individual mouse. *Figure S4: Detection of target derived peptides with recombinant NOX.* C4P, LA5P, CSP were spotted onto nitrocellulose membrane. Following blocking (with 2%BSA) the membrane was incubated with rNOX. rNOX was detected with rabbit anti rNOX antibodies and finally with goat anti rabbit HRP antibody.
(DOC)
