# Correction: NADH Oxidase Functions as an Adhesin in *Streptococcus pneumoniae* and Elicits a Protective Immune Response in Mice

**DOI:** 10.1371/annotation/7fbc524a-4035-401e-8cc7-93393afc35fd

**Published:** 2013-06-21

**Authors:** Lena Muchnik, Asad Adawi, Ariel Ohayon, Shahar Dotan, Itai Malka, Shalhevet Azriel, Marilou Shagan, Maxim Portnoi, Daniel Kafka, Hannie Nahmani, Angel Porgador, Johnatan M. Gershoni, Donald A. Morrison, Andrea Mitchell, Michael Tal, Ronald Ellis, Ron Dagan, Yaffa Mizrachi Nebenzahl

The name of the 12th author was spelled incorrectly. The correct name is: Jonathan M. Gershoni. 

