# Supplementary figures and images for: NADH Oxidase Functions as an Adhesin in Streptococcus pneumoniae and Elicits a Protective Immune Response in Mice
Source: PLoS One. 2013 Apr 8;8(4):e61128. doi: 10.1371/journal.pone.0061128 (PMC3620118; doi:10.1371/journal.pone.0061128)

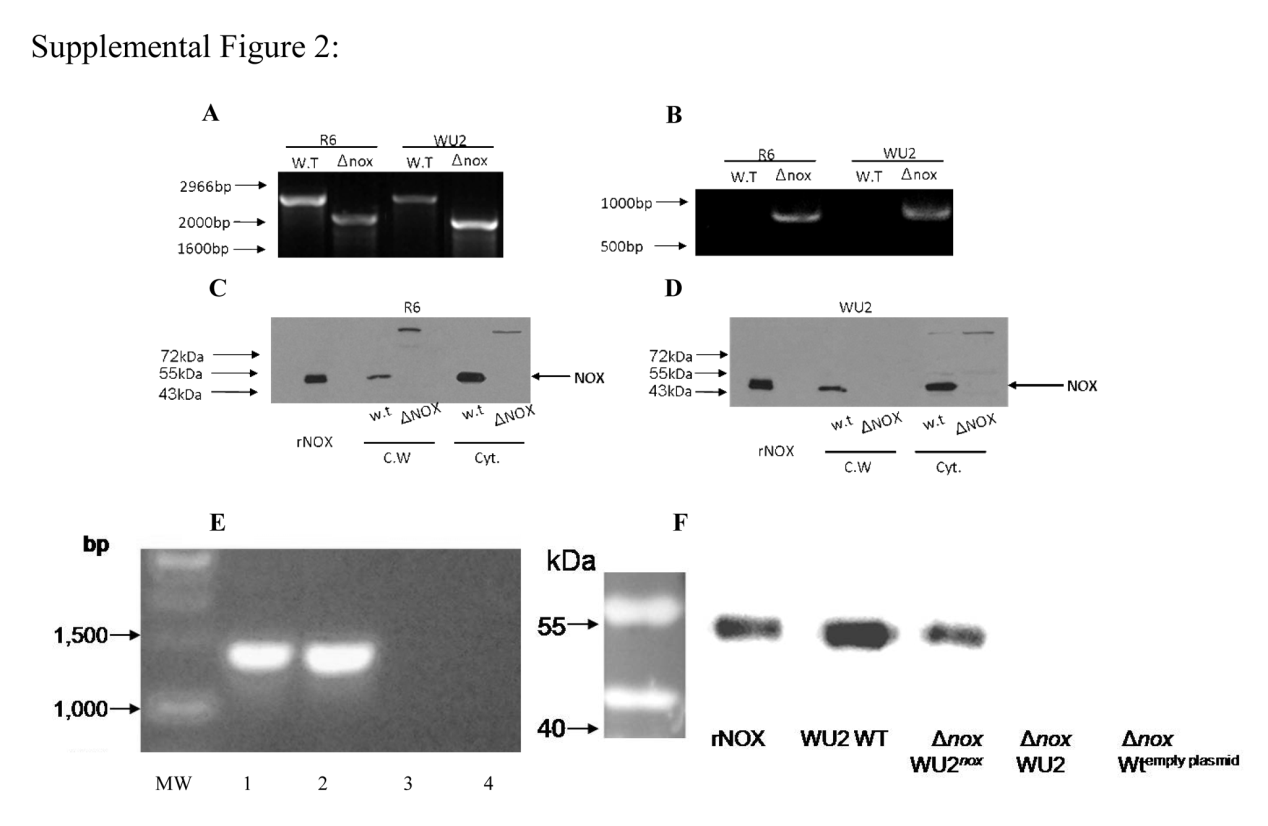
**Figure S1**

**Figure S2**

**
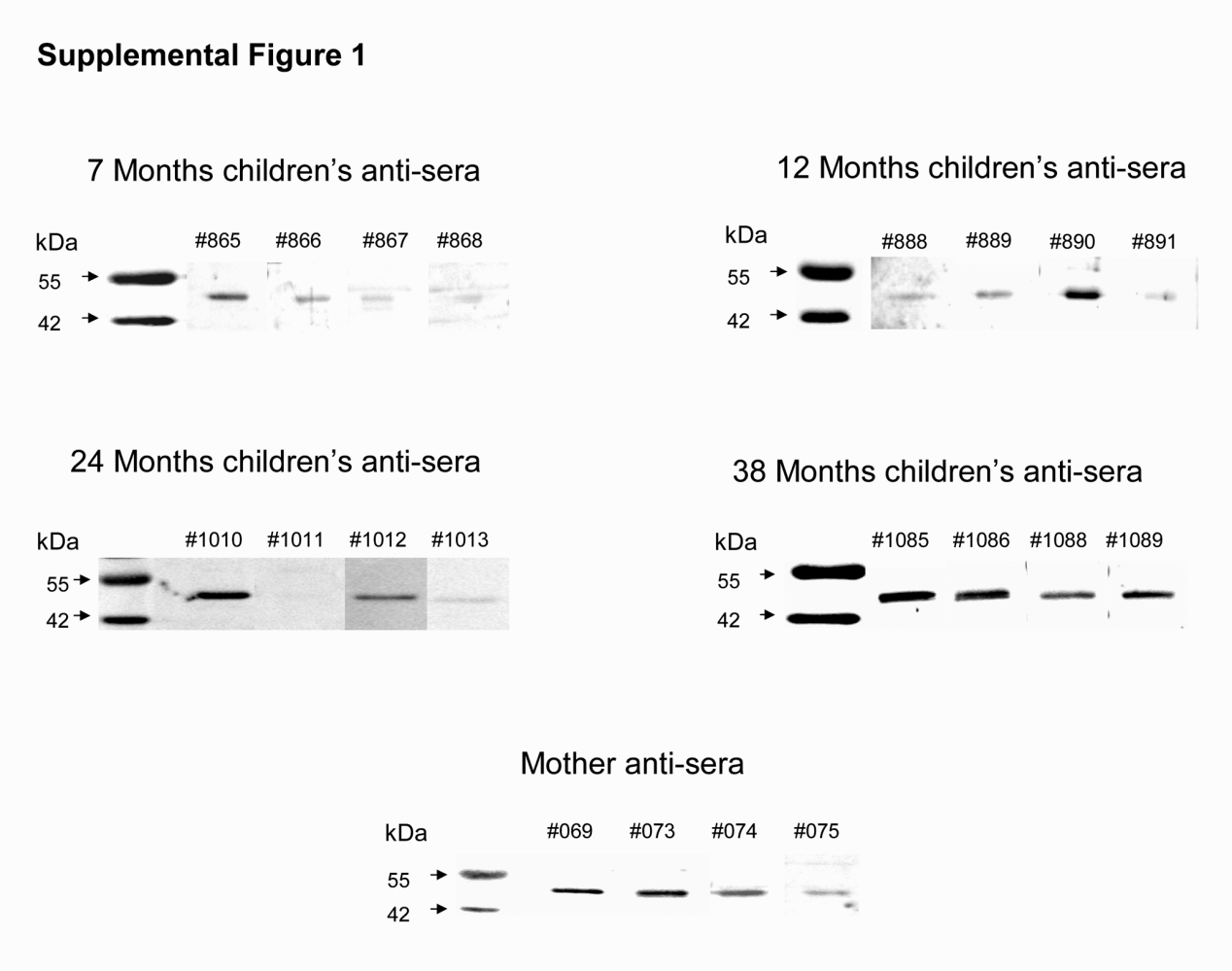
**

**Figure S3**


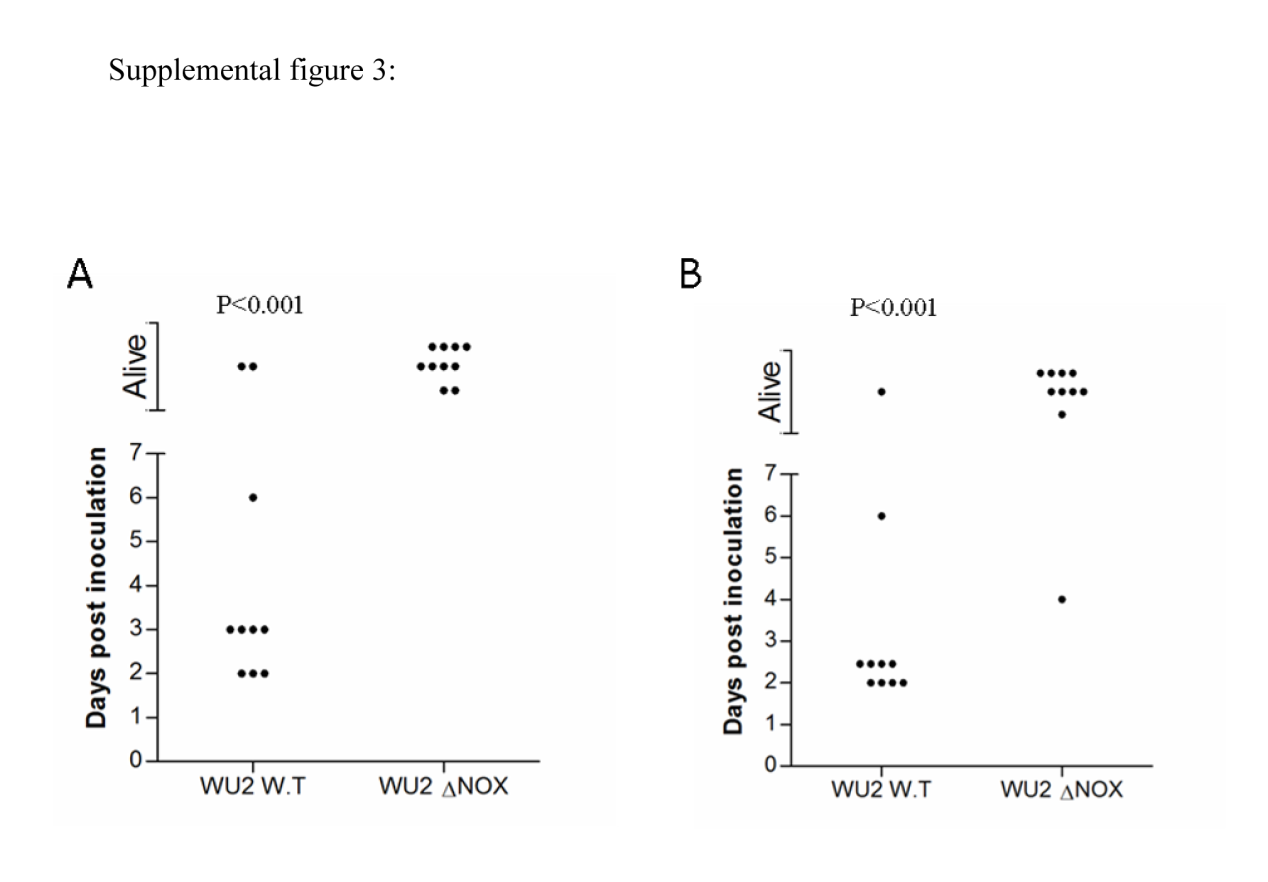


**Figure S4**


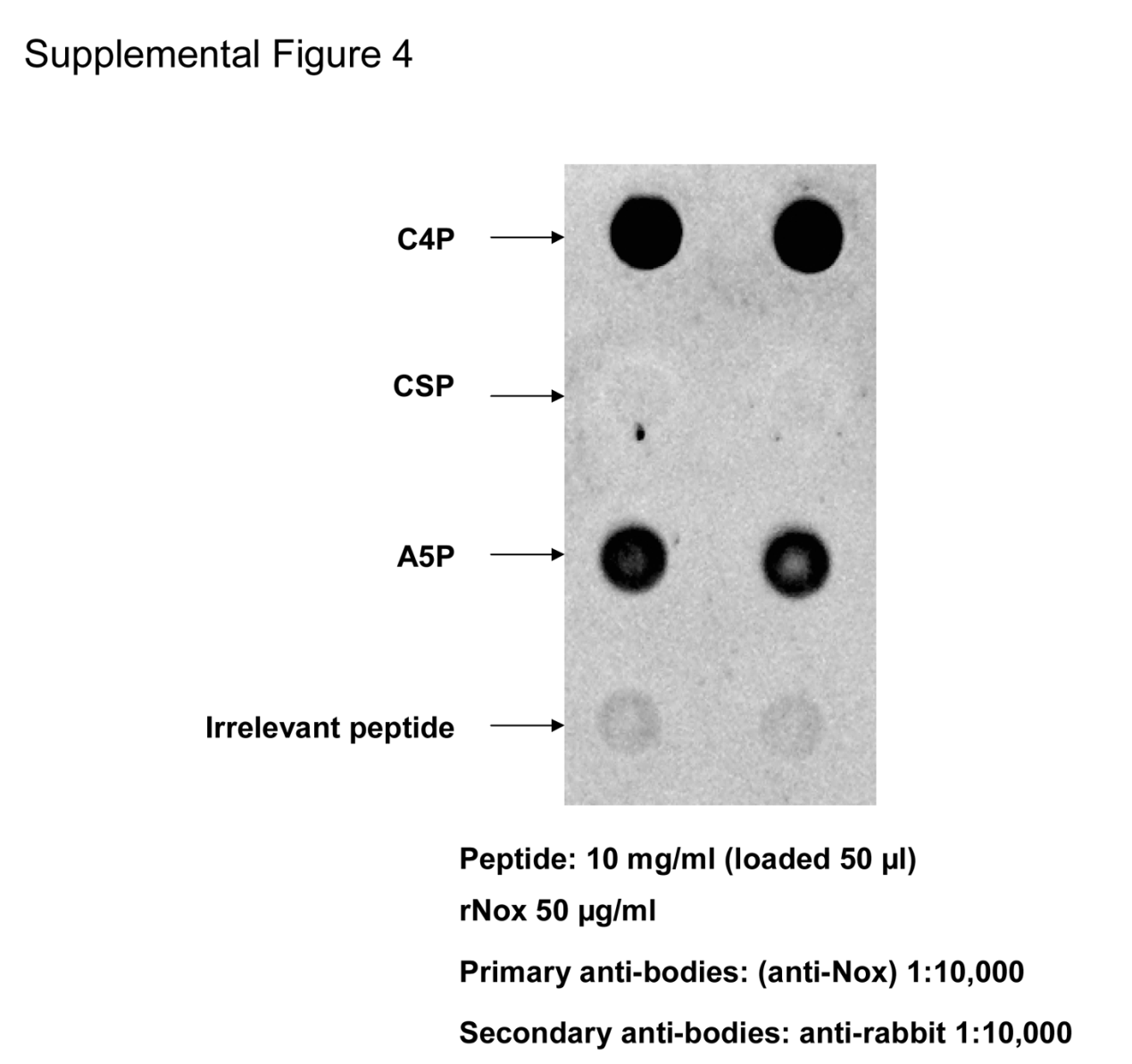

Supplement: File S1 — Supporting information Figures. Figure S1: Age dependent antigenicity to NOX in infants and children. In attempt to analyze the antibody levels against NOX in children at different ages, rNOX (10 ng) was separated on SDS PAGE, transferred to nitrocellulose membrane. The lanes on the nitrocellulos were cut and probed with sera obtained from different children at 7, 12, 24, and 38 months of age and 4 sera were obtained from random mothers. Sera were diluted 1∶20, the incubation was for the same duration and all the strips were developed together for 20 seconds. The human sera were detected with Goat anti-human IgG-HRP antibody. Figure S2: Production of null mutant bacteria and its supplementation. The transformation procedure was performed by homologous recombination as previously described [28]. The genomic DNA of the wild type R6, Δnox R6, WU2 and Δnox WU2 were amplified with the 3′ downwing (IM1) and 5′ upwing (IM4) primers (A) or with primers for erythromycin (B). C and D. Cytoplasmic and cell-wall fractions from R6 WT, Δnox R6 (C), WU2 WT and Δnox WU2 (D) were immunoblotted and probed with rabbit anti-rNOX antibodies. CW: Cell-wall fraction, Cyt: cytoplasmic fraction E. nox gene was amplified with primers for nox from: WT WU2 (lane 1), Δnox WU2nox (lane 2) lane 3 Δnox WU2empty plasmid (lane 3) and Δnox WU2 (lane 4). F. S. pneumoniae WU2 WT, Δnox WU2nox, Δnox WU2 and Δnox WU2empty plasmid were lysed and and NOX expression or lack of its expression was verified with rabbit anti NOX antibodies. Figure S3: Reduced virulence of NOX null mutant bacteria. A. Seven to 9 week old CBA/Nxid mice were inoculated IN (5×10^5^ CFU) with either WT WU2 (n = 10) or Δnox WU2 (n = 10) B. Seven to 9 week old CBA/Nxid mice were inoculated IP with either WT WU2 (n = 10; 64 CFU) or Δnox WU2 (n = 10, 70 CFU). The groups were matched for age. Survival was monitored daily; each point on the scatter plots represents an individual mouse. Figure S4: Detection of target derived peptides with rec [file pone.0061128.s001.doc]
